# Tumor cell-specific AIM2 regulates growth and invasion of cutaneous squamous cell carcinoma

**DOI:** 10.18632/oncotarget.17573

**Published:** 2017-05-02

**Authors:** Mehdi Farshchian, Liisa Nissinen, Elina Siljamäki, Pilvi Riihilä, Minna Piipponen, Atte Kivisaari, Markku Kallajoki, Reidar Grénman, Juha Peltonen, Sirkku Peltonen, Koen D. Quint, Jan Nico Bouwes Bavinck, Veli-Matti Kähäri

**Affiliations:** ^1^ Department of Dermatology, University of Turku and Turku University Hospital, Turku, Finland; ^2^ MediCity Research Laboratory, University of Turku, Turku, Finland; ^3^ Department of Pathology, University of Turku and Turku University Hospital, Turku, Finland; ^4^ Department of Otorhinolaryngology - Head and Neck Surgery, University of Turku and Turku University Hospital, Turku, Finland; ^5^ Department of Cell Biology and Anatomy, University of Turku, Turku, Finland; ^6^ Department of Dermatology, Leiden University Medical Center, Leiden, The Netherlands; ^7^ DDL Diagnostic Laboratory, Rijswijk, The Netherlands

**Keywords:** skin, cancer, inflammasome, AIM2, keratinocyte

## Abstract

Cutaneous squamous cell carcinoma (cSCC) is the most common metastatic skin cancer. Inflammation is a typical feature in cSCC progression. Analysis of the expression of inflammasome components in cSCC cell lines and normal human epidermal keratinocytes revealed upregulation of the expression of AIM2 mRNA and protein in cSCC cells. Elevated levels of AIM2 mRNA were noted in cSCCs *in vivo* compared with normal skin. Strong and moderate tumor cell specific expression of AIM2 was detected with immunohistochemistry (IHC) in sporadic human cSCCs *in vivo*, whereas expression of AIM2 was moderate in cSCC *in situ* (cSCCIS) and low or absent in actinic keratosis (AK) and normal skin. IHC of cSCCs, cSCCIS and AKs from organ transplant recipients also revealed strong and moderate tumor cell specific expression of AIM2 in cSCCs. Knockdown of AIM2 resulted in reduction in viability of cSCC cells and onset of apoptosis. RNA-seq and pathway analysis after knockdown of AIM2 in cSCC cells revealed downregulation of the biofunction category *Cell cycle* and upregulation of the biofunction category *Cell Death and Survival*. Knockdown of AIM2 also resulted in reduction in invasion of cSCC cells and downregulation in production of invasion proteinases MMP1 and MMP13. Knockdown of AIM2 resulted in suppression of growth and vascularization of cSCC xenografts *in vivo*. These results provide evidence for the role of AIM2 in the progression of cSCC and identify AIM2 inflammasome function as a potential therapeutic target in these invasive and metastatic tumors.

## INTRODUCTION

Keratinocyte-derived cutaneous squamous cell carcinoma (cSCC) is the most common metastatic skin cancer and its incidence is increasing due to aging of population and increased recreational exposure to the sunlight [1–3]. Cumulative lifetime sun exposure is an important risk factor for cSCC and other risk factors include chronic ulcers, chronic inflammation of the skin, and immunosuppression [4, 5]. The progression of actinic keratosis (AK) to cSCC *in situ* (cSCCIS) and invasive cSCC is associated with inflammation [6]. Changes in the microenvironment of the premalignant skin lesion, such as alteration of the composition of the epidermal basement membrane and dermal extracellular matrix, and accumulation of inflammatory cells and microbial structures, are possible mechanisms for the role of inflammation in progression of AK to cSCC [7]. On the other hand, cSCCs in immunosuppressed patients progress rapidly and have been reported to be associated with higher rate of recurrence, metastasis, and mortality [8–10].

Inflammasomes are important components of the innate immune response involved in onset of inflammation. Inflammasomes serve as sensors for exogenous and endogenous danger signals and trigger activation and secretion of interleukin (IL)-1β and IL-18 [11]. Inflammasomes contain 1) a scaffold and sensor protein, either a Nod-like receptor (NLRP1, NLRP3, NLRC4, and NLRP6), or a HIN (hematopoietic IFN inducible nuclear antigen) domain protein, AIM2 (absent in melanoma 2) or IFI16 (IFN-γ-inducible protein 16), 2) adaptor protein ASC (apoptosis associated speck-like protein containing a CARD), and 3) effector protein caspase-1 [12]. The HIN-200 domain of AIM2 and IFI16 serves as a sensor for cytoplasmic double stranded DNA and the pyrin domain interacts with ASC for activation of caspase-1 [13, 14]. Inflammasome function has mainly been characterized in immune cells, but NLRP1, NLRP3, and AIM2 inflammasomes have also been found in epidermal keratinocytes [15]. AIM2 inflammasome has been shown to be involved in the pathogenesis of autoimmune disorders, including psoriasis and systemic lupus erythematosus [13, 16, 17]. In addition, the role of inflammasome activation in autoinflammatory disorders has recently been emphasized [15].

Here, we have examined the role of inflammasome in the progression of cSCC. We show, that the expression of AIM2 is specifically upregulated in cSCC cells in culture and in tumor cells in cSCCs of immunocompetent individuals and organ transplant recipients (OTRs) *in vivo*. In addition, AIM2 knockdown results in reduction in cSCC cell viability and invasion, and suppression of growth and vascularization of cSCC xenografts *in vivo*. These results provide evidence for the role of AIM2 in the progression of cSCC and identify AIM2 inflammasome function as a potential therapeutic target in recurrent and metastatic cSCCs.

## RESULTS

### Upregulation of AIM2 expression in cSCC cells

**Figure 1:**
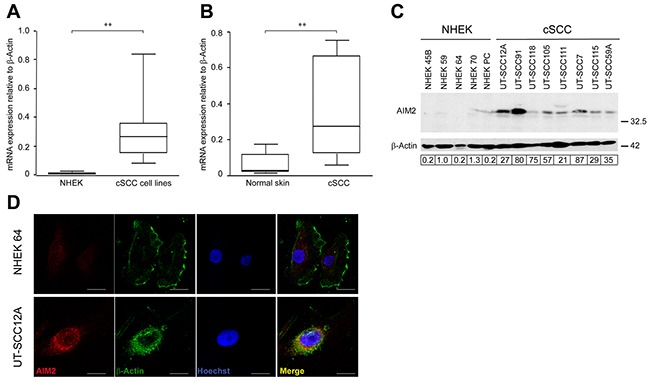
Upregulation of AIM2 expression in cSCC cells **(A)** *AIM2* mRNA levels in primary (n=5) and metastatic (n=3) human cSCC cell lines and in NHEKs (n=5) were determined with qRT-PCR. **(B)** *AIM2* mRNA levels in cSCC tumors (n=6) and normal human skin (n=6) *in vivo* were analyzed by qRT-PCR. **(C)** AIM2 protein levels in cell lysates of NHEKs and cSCC cells were analyzed by Western blotting with β-actin as a marker for loading. Level of AIM2 was quantitated by densitometry and corrected for the level of β-actin. **(D)** Indirect immunofluorescence staining was used to localize AIM2 and β-Actin in the cSCC cells and NHEKs. Nuclei were visualized with Hoechst staining. Scale bar=10 μm. (**P<0.01; Mann-Whitney two-way *U*-test).

The expression of mRNAs for inflammasome components in primary (n=5) and metastatic (n=3) cSCC cell lines and normal human epidermal keratinocytes (NHEKs) from 5 individuals was analyzed using RNA-seq. The expression of molecules involved in pathways related to innate immunity (KEGG pathways: Cytosolic DNA-sensing, NOD-like receptor signaling, RIG-I-like receptor signaling) in NHEKs and cSCC cells is shown in Supplementary Figure 1. These results showed specific upregulation of *AIM2* mRNA expression in cSCC cells, as compared to NHEKs (Supplementary Figure 1A, 1B). Significantly elevated levels of *AIM2* mRNA were also noted in cSCC cell lines with quantitative real-time PCR (qRT-PCR), whereas the expression level was very low in NHEKs (Figure 1A). The mean level of *AIM2* mRNA expression was also significantly higher in RNA from cSCC tumors (n=6) compared with normal skin (n=6) *in vivo* (Figure 1B).

Production of AIM2 by cSCC cell lines and NHEKs was analyzed by Western blotting of the cell lysates. A specific 38 kDa band corresponding to AIM2 was detected in all cSCC cell lines, whereas production of AIM2 protein in NHEKs was very low (Figure 1C). Immunofluorescence (IF) staining of permeabilized cSCC cells and NHEKs for AIM2 revealed abundant perinuclear expression of AIM2 in cSCC cells, whereas only weak labeling for AIM2 was noted in NHEKs (Figure 1D).

No significant difference in *IFI16* mRNA levels was noted between cSCC cell lines and NHEKs (Supplementary Figure 2A), or between cSCC tumors *in vivo* and normal skin by qRT-PCR (Supplementary Figure 2B). Production of IFI16, adaptor protein ASC and caspase-1 was noted both in NHEKs and in cSCC cells (Supplementary Figure 2C). Based on the specific upregulation of AIM2 expression in cSCC cells, it was selected for further characterization in cSCC.

### Overexpression of AIM2 by tumor cells in sporadic and organ transplant recipients cSCCs *in vivo*

**Figure 2:**
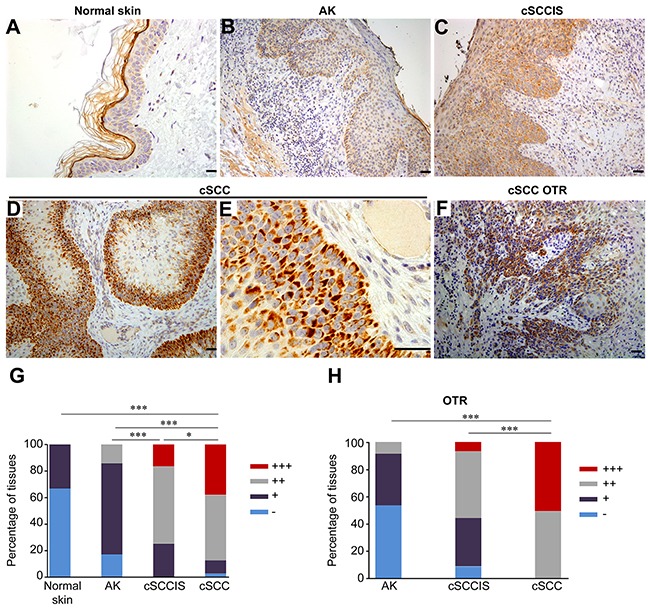
Expression of AIM2 by tumor cells in sporadic and organ transplant recipient (OTR) cSCCs *in vivo* **(A-E)** AIM2 expression was determined by IHC of TMAs containing normal human skin (n=15), actinic keratosis (AK) (n=71), cSCCs *in situ* (cSCCIS) (n=60) and sporadic cSCCs (n=81). In sporadic, UV-induced human cSCC tumor cell-specific cytoplasmic and perinuclear localization of AIM2 was detected and the expression level was mainly strong (+++) **(D, E)** or moderate (++). In cSCCIS, AIM2 expression level was moderate (++) **(C)** in the majority of sections. In AK, AIM2 expression was mainly weak (+) **(B)**. AIM2 expression was absent (−) or weak (+) **(A)** in normal skin samples. **(F)** Tumor cell-specific cytoplasmic and perinuclear expression of AIM2 was detected in cSCCs of OTRs. Scale bar=50 μm. **(G)** AIM2 expression level was significantly stronger in sporadic cSCCs compared with cSCCIS, AK and normal skin. **(H)** In OTR derived tissues, AIM2 expression was significantly more abundant in cSCC (n=57) compared with cSCCIS (n=59) and AK (n=58). (*P<0.05, *** *P*<0.001; Fisher's exact test).

To analyze the expression of AIM2 *in vivo*, tissue microarrays (TMAs) consisting of normal skin (n=15), UV-induced premalignant lesions (AK) (n=71), cSCCIS (n=60), and cSCCs (n=81) were analyzed by immunohistochemistry (IHC). In normal skin, expression of AIM2 was absent (−) (67%) or weak (+) (33%) (Figure 2A, 2G). Epidermal layer of AK was mainly weakly positive (+) for AIM2 in the majority of samples (69%) (Figure 2B, 2G). In cSCCIS, cytoplasmic AIM2 expression level was mainly moderate (++) (58%) (Figure 2C, 2G). In invasive cSCCs, cytoplasmic and perinuclear AIM2 was noted in tumor cells in the invasive margin and the expression was mainly strong (+++) (38%) (Figure 2D, 2E, 2G) or moderate (49%). AIM2 expressing tumor cells were located at the invasive edges of the cSCC tumor (Supplementary Figure 3). In whole tumor sections of cSCC, AIM2 expression was seen in tumor cells, but the adjacent normal skin was negative or weak for AIM2 (Supplementary Figure 4). Semiquantitative analysis revealed significantly more abundant AIM2 expression in sporadic cSCCs compared with cSCCIS, AKs and normal skin (Figure 2G). In addition, AIM2 expression in cSCCIS was significantly more abundant than in AKs (Figure 2G). AIM2 expression was also analyzed in whole tumor sections of AK (n=58), cSCCIS (n=59) and cSCC (n=57) of OTRs by IHC. Tumor cell-specific cytoplasmic and perinuclear AIM2 expression was noted in all cSCCs (Figure 2F). Semiquantitative analysis of the AIM2 expression (Figure 2H) revealed strong (+++) (Figure 2F) (51%) and moderate (++) (49%) AIM2 expression in cSCC of OTRs. AIM2 expression level was significantly stronger in cSCC compared with cSCCIS and AK in OTRs (Figure 2H).

### Alteration of gene expression profile in cSCC cells after AIM2 knockdown

**Figure 3:**
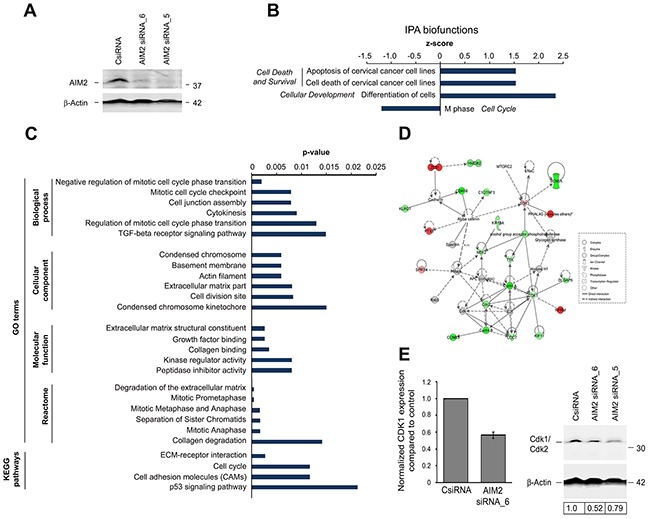
Alteration of gene expression profile in cSCC cells afterAIM2 knockdown **(A)** cSCC cells (UT-SCC7) were transfected with AIM2 siRNA_5, AIM2 siRNA_6, or control siRNA (CsiRNA) (75 nM) and transfection efficiency was examined by Western blotting 72 hours after transfection. β–Actin was used as a marker for equal loading. **(B-E)** Three cSCC cell lines (UT-SCC7, UT-SCC12A, and UT-SCC105) were transfected with AIM2 siRNA_6 or control siRNA. 72 hours after transfection, whole transcriptome analysis was performed with RNA-seq. Summary of **(B)** Ingenuity pathway analysis (IPA) biofunctions and **(C)** gene ontology (GO) terms and Kyoto Encyclopedia of Genes and Genomes (KEGG) pathways related to AIM2 knockdown (P < 0.05, FC log2 > 0.75). **(D)** Molecular interactions involved in the network *Cell cycle, cellular assembly and organization, DNA replication, recombination, and repair* as one of the top networks regulated after AIM2 knockdown in cSCC cells (score=24; Supplementary Table 1) are depicted with IPA (*P*<0.05 and fold change (FC) (log2) >0.75). Red color indicates up-regulation, and green color indicates down-regulation. The intensity of the color shows the magnitude of FC. The arrows and the lines show the interactions (solid line indicated direct and dashed line indirect interaction). **(E)** Normalized CDK1 mRNA expression was determined from cSCC cells with RNA-seq after AIM2 siRNA_6 knockdown (left panel). UT-SCC7 cells were transfected with AIM2 siRNA_5, AIM2 siRNA_6, or control siRNA (75 nM) and expression of Cdk1/Cdk2 was determined by Western blotting 72 hours after transfection with siRNAs. Level of Cdk1/Cdk2 was quantitated by densitometry and corrected for the level of β–Actin used as a marker for equal loading (right panel).

To examine the functional role of AIM2 in cSCC cells, two specific siRNAs were used to knock down the expression of AIM2 (Figure 3A). RNA-seq analysis was performed after AIM2 knockdown of three cSCC cell lines. Pathway analysis of the genes significantly regulated following AIM2 knockdown revealed significant downregulation of the biofunction category *Cell Cycle* and upregulation of biofunction category *Cell Death and Survival* (Figure 3B). Among the top molecular networks regulated after AIM2 knockdown were *Cell Death and Survival, Cellular Development, Organismal Development* (score=32) and *Cell cycle, cellular assembly and organization, DNA replication, recombination, and repair* (score=24; Supplementary Table 1). In addition, the genes significantly regulated following AIM2 knockdown were associated with GO terms including *Degradation of extracellular matrix* and KEGG pathways like *ECM-receptor interaction* (Figure 3C). Analysis of *Cell cycle, cellular assembly and organization, DNA replication, recombination, and repair* as one of the top molecular networks regulated after AIM2 knockdown revealed downregulation of the expression of several cell cycle related genes: *CDK1*, *CDC7, CCNA1, CCNB3*, *KIF11*, and *TTK* (Figure 3D). Cdk1 is a key player in cell cycle regulation, which has been shown to be able to bind all mammalian cyclins and to be sufficient to drive cell cycle [18]. Downregulation of Cdk1 after AIM2 siRNA knockdown was detected both at mRNA and protein level (Figure 3E).

### Knockdown of AIM2 results in reduced cell viability and invasion

**Figure 4:**
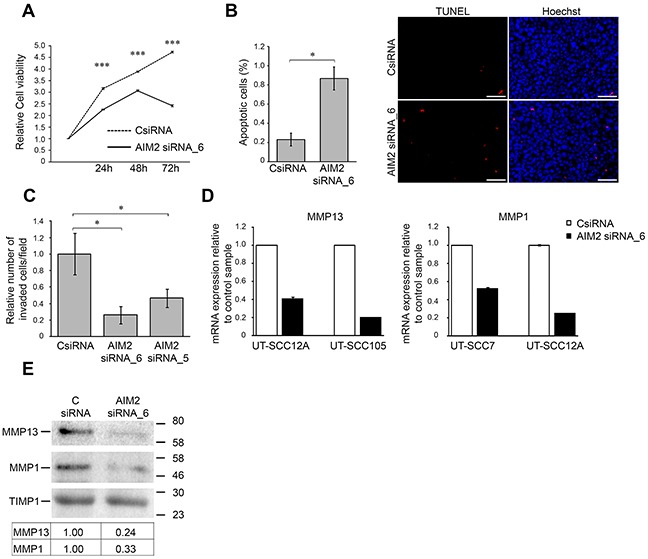
Knockdown of AIM2 inhibits viability and invasion of cSCC cells **(A)** The number of viable cSCC cells (UT-SCC7) was determined 24, 48, and 72 hours after transfection with AIM2 siRNA_6 and control siRNA (CSiRNA) (75 nM) (mean ± SD, n=8). **(B)** cSCC cells (UT-SCC7) were transfected with AIM2 siRNA_6, or control siRNA and 48 hours after transfection apoptotic cells were detected with TUNEL staining and the relative number of TUNEL positive apoptotic cells with positive nuclear staining was determined by counting 2000-3000 cells at 10× magnification per image (n=3, mean ± SD) (left panel). Representative images of the quantification are shown. Scale bar=100 μm (right panel). **(C)** UT-SCC7 cell lines were transfected with AIM2 or control siRNAs and seeded to the inserts coated with type I collagen 24 hours after transfection. After 48 hours the number of invaded cells was counted (mean ± SD, n=3). **(D)** MMP13 and MMP1 mRNA levels were determined by qRT-PCR 72 hours after AIM2 knockdown in cSCC cells. **(E)** Levels of MMP13 and MMP1 in conditioned media of cSCC cells (UT-SCC12A) were determined by Western blot analysis 72 hours after AIM2 knockdown. TIMP1 was used as a marker for equal loading. Levels of MMP1 and MMP13 were quantitated by densitometry and corrected for the level of TIMP1. (*P<0.05, ***P<0.001; Student's *t*-test).

To further explore the functional role of AIM2 in cSCC cells, expression of AIM2 was knocked down with two specific siRNAs (Figure 3A). Analysis of cell viability revealed significant reduction in the number of viable cSCC cells 24, 48, and 72 hours after transfection with AIM2 siRNA compared with control siRNA transfected cells (Figure 4A and Supplementary Figure 5). The experiment was repeated with three different cSCC cells lines with similar results (data not shown). In addition, the number of cSCC cells was significantly decreased 72 hours after AIM2 siRNA transfection, as compared with the cells transfected with control siRNA (Supplementary Figure 6). Increased number of TUNEL positive cells, as a sign for apoptosis was also detected 48 hours after AIM2 siRNA transfection, as compared with control siRNA transfected cells (Figure 4B). Potent inhibition of cSCC cell invasion through collagen was noted 72 hours after transfection of the cSCC cells with the AIM2 siRNA, as compared with the control siRNA transfected cSCC cells (Figure 4C). This experiment was repeated with two cSCC cells lines with similar results (data not shown). Production of MMP13 and MMP1, two collagenolytic invasion proteinases associated with the invasion of cSCC cells, was notably inhibited 72 hours after AIM2 knockdown in cSCC cells both at mRNA (Figure 4D) and protein level (Figure 4E and Supplementary Figure 7).

### Knockdown of AIM2 results in suppression in growth and vascularization of human cSCC xenografts

**Figure 5:**
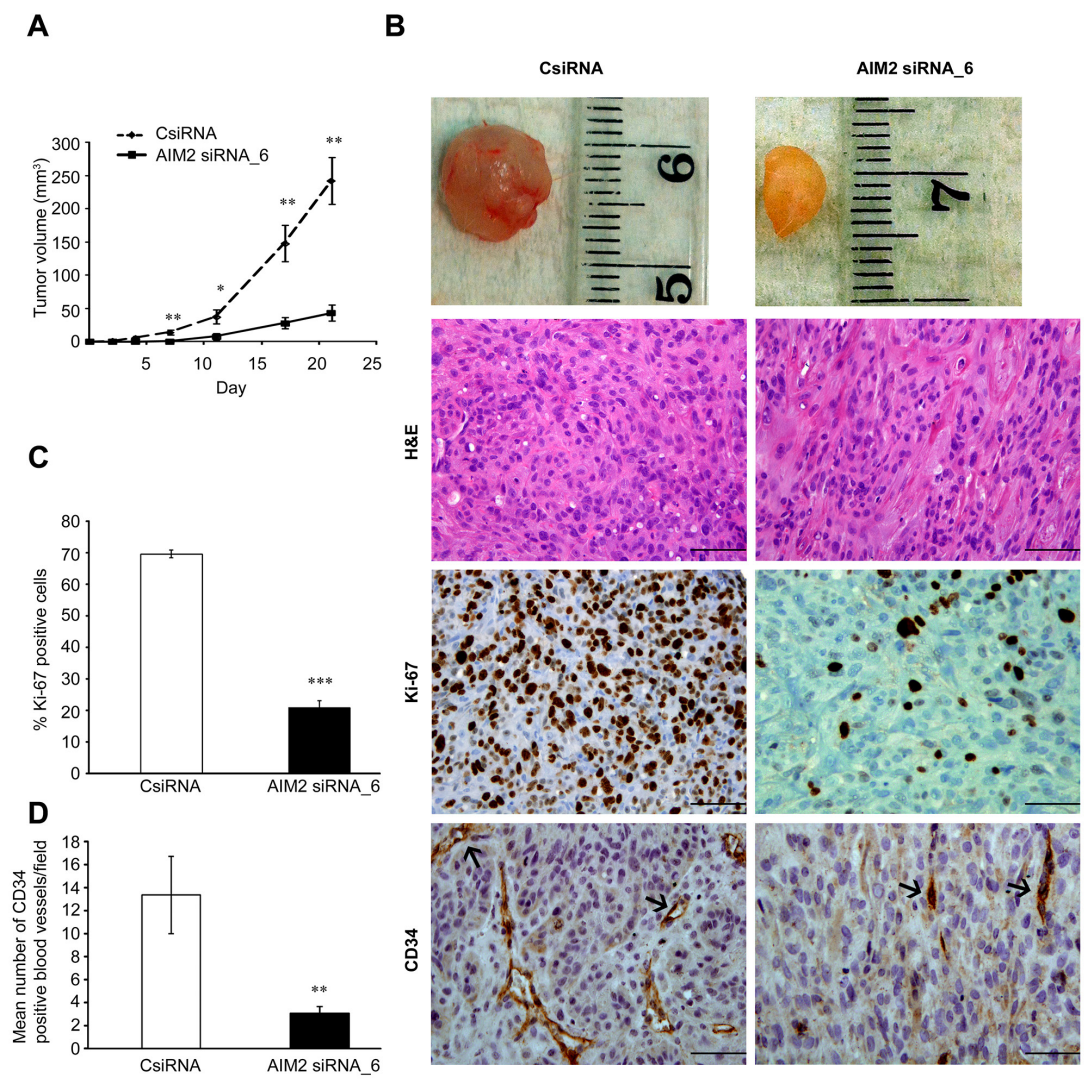
AIM2 knockdown suppresses growth and vascularization of human cSCC xenografts **(A)** cSCC cells (UT-SCC7) were transfected with AIM2 siRNA_6 or control siRNA (75 nM). 72 hours after transfection, cells (5×10^6^) were injected subcutaneously into the back of SCID mice (n=8 for each group) and the size of tumors was measured twice a week (mean ± SEM). **(B)** Tumors were harvested after 21 days, embedded in paraffin and stained with H&E (upper panels), for proliferation marker Ki-67 (middle panels), and for vascular endothelial marker CD34 (lower panels). **(C)** The percentages of Ki-67-positive tumor cells in xenografts were counted (mean ± SEM). **(D)** The density of CD34-positive blood vessels in xenografts was determined in each tumor. Scale bar=100μm (*P<0.05, **P<0.01, ***P<0.001; Student's *t*-test).

The role of AIM2 in growth of the cSCC tumors *in vivo* was examined in xenograft model. Metastatic cSCC cells (UT-SCC7) (5×10^6^) were transfected with AIM2 siRNA or control siRNA, incubated for 72 hours and injected subcutaneously into the back of SCID mice. Significant suppression in growth of the xenograft tumors was noted in AIM2 siRNA group compared with the control siRNA group (Figure 5A, 5B). Histological analysis of the xenografts harvested 21 days after the implantation revealed, that tumors in AIM2 siRNA group were less cellular compared with xenografts in control siRNA group (Figure 5B). IHC analysis of the tumors for proliferation marker Ki-67 showed significantly lower number of proliferating cells in the xenografts established with cSCC cells transfected with AIM2 siRNA (21%) compared with control siRNA tumors (70%) (Figure 5B, 5C). Analysis of the vasculature of the xenografts using IHC for vascular marker CD34 revealed significantly reduced number of CD34-positive blood vessels in AIM2 knockdown tumors compared with the controls (Figure 5B, D).

## DISCUSSION

There is increasing evidence that chronic inflammation is associated with cancer progression [19, 20]. Chronic inflammation has been recognized as a risk factor for cSCC [4] and inflammation is a typical feature of the progression of AK lesions to invasive and metastatic cSCC [6]. Inflammasomes are important components of the innate immune response involved in the onset of inflammation. Inflammasome component AIM2 serves as a sensor for cytoplasmic double-stranded DNA, and this way plays a key role in response to bacterial and viral colonization [21]. Activation of inflammasome by cytoplasmic DNA in epidermal keratinocytes can promote initiation of inflammation in autoimmune and autoinflammatory skin diseases [13, 15, 22].

There is recent evidence for the involvement of AIM2 in carcinogenesis, but the exact role of AIM2 in cancer progression remains unclear. In colorectal cancer AIM2 appears to play a protective role as lack of AIM2 expression is associated with cancer progression, metastasis, and less favorable prognosis [23]. On the other hand, restoration of AIM2 has been shown to stimulate invasion of colorectal cancer cells [24]. Overexpression of AIM2 has been shown to suppress proliferation of breast cancer cells and growth of xenografts [25]. Elevated expression level of AIM2 in senescent prostate epithelial cells is associated with increased IL-1β production and development of benign prostatic hyperplasia, whereas the expression of AIM2 in prostate cancer cells is low [26].

Here, we have examined the role of AIM2 in the progression of cSCC. Our results show that the expression of AIM2 is specifically upregulated in primary and metastatic cSCC cell lines compared with NHEKs using RNA-seq technique and qRT-PCR. Analysis of the AIM2 mRNA expression level by qRT-PCR revealed upregulation of AIM2 mRNA also in cSCC tumors *in vivo* compared with normal skin. In addition, markedly higher level of AIM2 protein in cSCC cell lines compared with NHEKs were detected with Western blot analysis and IF staining.

Analysis of a large panel of tissue samples from normal skin, AK, cSCCIS, and sporadic cSCCs by IHC revealed tumor cell specific expression for AIM2 in cSCCs. In accordance with previous observations, the cellular localization of AIM2 was cytoplasmic and perinuclear [27, 28]. In the majority of cSCCs, the level of AIM2 expression was strong or moderate and although the majority of the cSCCIS lesions showed moderate AIM2 expression, a trend towards strong expression was noted. Expression of AIM2 was mainly weak or absent in AKs and normal skin. Analysis of cSCCs, cSCCIS and AKs of OTRs with IHC revealed strong and moderate tumor cell specific AIM2 expression in cSCCs. The majority of AK lesions of OTRs were negative for AIM2, whereas in cSCCIS the expression of AIM2 was mainly moderate or weak. Altogether, these results show that the expression of AIM2 is very low in normal intact skin and that it is specifically induced in tumor cells in cSCCs suggesting AIM2 as a biomarker for the progression of premalignant lesions toward invasive cSCC.

Knockdown of AIM2 expression in cSCC cells resulted in significant suppression in the growth of cSCC xenografts compared with tumors in the control siRNA group. Furthermore, AIM2 knockdown resulted in marked reduction in vascularization of the xenografts. Together these findings provide evidence for the role of AIM2 in cSCC growth. Functional studies with cSCC cells showed, that knockdown of AIM2 resulted in reduction in viability and increase in the number of apoptotic cells. Furthermore, silencing of AIM2 expression resulted in decreased invasion of cSCC cells in culture associated with reduction in the production of MMP13 and MMP1, two collagenolytic MMPs associated with cSCC invasion [29, 30]. It is therefore possible, that reduction in MMP13 and MMP1 production after AIM2 knockdown impairs implantation of cSCC cells in the skin of SCID mice and this way results in delay in tumor growth.

The molecular basis for the role of AIM2 in the growth of cSCCs was elucidated by RNA-seq analysis of cSCC cells after AIM2 knockdown. Pathway analysis of the gene expression profile following AIM2 knockdown revealed significant downregulation of the biofunction *M phase* in the category *Cell Cycle* and upregulation of the biofunction related to category *Cell Death and Survival*. In addition, downregulation of the expression of several genes coding for proteins regulating cell cycle, including *CDK1*, *CDC7, CCNA1, CCNB3*, *KIF11*, and *TTK* in the network *Cell cycle, cellular assembly and organization, DNA replication, recombination, and repair*, was noted following AIM2 knockdown. Cdk1 is a key player in cell cycle regulation and has been shown to be sufficient to drive cell cycle [18]. These observations provide mechanistic evidence, that AIM2 is involved in regulation of the viability of cSCC cells and may this way promote growth of cSCC. It is possible, that this effect is mediated indirectly by cytokines released by cSCC cells in response to activation of AIM2 inflammasome. Further studies using AIM2 knock-in are required to elucidate the biological function of AIM2 in cSCC cell growth.

Taken together, our findings revealed specific upregulation of AIM2 expression in cSCC cells in culture and *in vivo*. These results are in accordance with a recent study reporting AIM2 expression in a limited number of cSCCs [31]. Our results also elucidated the role of AIM2 in the progression of sporadic cSCCs and cSCCs in OTRs, a specific population of immunosuppressed patients at risk for developing multiple cSCCs [10]. Knockdown of AIM2 resulted in suppression of cSCC growth *in vivo* and in reduced viability and invasion of cSCC cells in culture. Small molecule inhibitors and antibodies are currently being studied to target inflammasome components for the cancer therapy [32]. The results of the present study suggest AIM2 as a putative therapeutic target in cSCCs, in patients with unresectable, recurrent, or multiple tumors, and in patients with high risk for cSCC, *e.g*. immunosuppressed patients.

## MATERIALS AND METHODS

### Ethical issues

Approval for use of tissue specimens and the collection of normal skin and SCC tissues was obtained from the Ethics Committee of the Hospital District of Southwest Finland, Turku, Finland and from the Leiden University Medical Center (LUMC), Leiden, the Netherlands. The study was performed in accordance with the ethical guidelines of the Declaration of Helsinki. Each patient gave their informed consent. The experiments with mice were approved by the State Provincial Office of Southern Finland and conducted according to institutional guidelines.

### Cell culture

Human cSCC cell lines (n=8) were established from surgically removed SCCs of skin [33]. Five cSCC cell lines were derived from primary cSCCs (UT-SCC12A, -91, -105, -111, and -118) and three from metastatic cSCCs (UT-SCC7, -59A, and -115). Authentication of the cell lines was performed by STR DNA profiling [33]. NHEKs were cultured from healthy skin of individuals undergoing surgery for mammoplasty (n=4) [34] or purchased from PromoCell (n=1) (Heidelberg, Germany). NHEKs and cSCC cells were cultured as previously described [35].

### Tissue samples

For RNA extraction, samples of cSCCs (n=6) were obtained in Turku University Hospital from surgery of primary tumors and samples of normal skin (n=6) were obtained from mammoplastic surgery [35]. TMAs consisting of samples from normal sun-protected skin (n=15), AK (n=71), cSCCIS (n=60), and UV-induced cSCC (n=81) were generated from the archival paraffin blocks of the Department of Pathology, Turku University Hospital [34–37]. Whole tumor sections of AK (n=58), cSCCIS (n=59) and cSCC (n=57) of immunosuppresed OTRs were obtained in the Department of Dermatology, Leiden University Medical Center, Leiden, Netherlands.

### Gene expression profiling

RNA and cDNA preparation were performed as previously described [35, 36]. Total RNA for RNA-seq was isolated from cells using miRNAeasy Mini kit (Qiagen, Chatsworth, CA). The Whole Transcriptome Analysis (SOLiD™, Applied Biosystems, Foster City, CA) was performed as described previously (accession number GSE66412) [37]. RNA-seq was performed with RNAs derived from cSCC cell lines (n=3) 72 hours after AIM2 siRNA transfection using Illumina RNA-sequencing (Illumina Inc., San Diego, CA, USA) at Finnish Microarray and Sequencing Centre, Turku Center for Biotechnology. The samples were sequenced with the HiSeq2500 instrument using single-end sequencing chemistry with 50 bp read length and aligned against the human reference genome (hg19 assembly). Pathway analysis was performed with Ingenuinity Pathway Analysis (Ingenuity Systems, Redwood City, Redwood City, CA). *P*<0.05 and fold change (log2) >0.75 were used as thresholds in data analysis. RNA-seq data (accession number GSE94270) have been deposited in the public database GEO (Gene Expression Omnibus, NCBI; http://www.ncbi.nlm.nih.gov/geo/).

### Quantitative RT-PCR

Levels of mRNA of AIM2, IFI16, MMP13 and MMP1 mRNA were determined with qRT-PCR using Applied Biosystems 7900HT Fast Real-Time PCR System [37, 38] and specific probes and primers (Oligomer, Finland) as follows:

*AIM2* forward primer 5′-GGCCCAGCAGGAA TCTATCAG-3′

*AIM2* reverse primer 5′-GAAGGGCTTCTTTGC TTTCAGTAC-3′

*AIM2* probe 5′-Fam-AAGGGTTTCAGAAGCGCT GTTTGCC-Tamra-3′

*IFI16* forward primer 5′- TCCTCAGATGCCTC-CATCAAC-3′

*IFI16* reverse primer 5′- CAGGTTCAGTCTTCA-GTCTTGGT-3′

*IFI16* probe 5′-Fam- CCAAGCAGCAGTTTCTT AACCACGT-Tamra-3′

Primers and probes for MMP13 and MMP1 have been described previously [36]. Level of β-actin mRNA was used as control [35].

### Western blot analysis

Production of AIM2 in cSCC cell lines and NHEKs was determined by Western blotting using anti-AIM2 antibody (Abnova, Walnut, CA, USA) as previously described [36]. Levels of MMP1 and MMP13 in conditioned media were determined by Western blotting using anti-MMP1 (Chemicon, Temecula, CA) and anti-MMP13 (Calbiochem) antibodies. Anti-β-actin (AC-15) (Sigma-Aldrich, St Louis, MO, USA) and anti-TIMP1 (Calbiochem) were used as the loading control [36, 37]. Levels of IFI16, casp-1, ASC, and Cdk1 were determined by Western blotting using anti-IFI16 (HPA002134) and anti-casp-1 (HPA003056) (both from Sigma, St. Louis, USA), anti-ASC (AB3607, Merck Millipore, Temecula, CA) and anti-Cdk1/Cdk2 (sc-53219, Santa Cruz Biotechnology, Santa Cruz, CA) antibodies.

### Immunofluorescence staining

Immunofluorescence staining was performed as previously described [37]. Briefly, cSCC cells were fixed with 3% paraformaldehyde and blocked with phosphate buffer saline (PBS) containing 3% bovine serum albumin (BSA). Cells were permeabilized with 0.1% Triton X-100 and labeled with anti-AIM2 antibody (Abnova). Highly cross-adsorbed Alexa Fluor® 568 goat anti-mouse IgG (H+L) (Invitrogen, Carlsbad, CA) was used as a secondary antibody, and Hoechst (H3570; Invitrogen) was used to visualize nuclei. The cells were examined with Zeiss LSM510 Meta confocal microscope (Zeiss, Jena, Germany), and the imaging was performed at the Cell Imaging Core, Turku Centre for Biotechnology, University of Turku and Åbo Akademi University.

### Immunohistochemistry

Automated immunostaining device (Ventana Medical Systems SA, Illkrich, France) was used to perform IHC of human TMAs and whole tumor sections of OTRs using anti-AIM2 antibody (Sigma, St. Louis, USA), 1:150 [37]. All slides were digitally scanned using a Pannoramic 250 Flash (3DHistech, Budapest, Hungary). AIM2 expression was assessed semiquantitatively by two observers (M.F. and L.N.) as negative (−), weak (+), moderate (++), and strong (+++).

### AIM2 knockdown with siRNA

cSCC cells were transfected with two different specific AIM2 siRNAs and control siRNA (75 nM) (Qiagen, Valencia, CA) using the silentFect^TM^ Lipid Reagent (BIO-RAD) [37]. The following sequence was targeted:

AIM2 siRNA_6: 5′-CCCGAAGATCAACACGCT TCA-3′

AIM2 siRNA_5: 5′AAAGGTTAATGTCCCGCT GAA-3′

Control siRNA: 5′-AATTCTCCGAACGTGTCA CGT-3′

### Cell viability assay

cSCC cells were transfected with control siRNA and AIM2 siRNAs (75nM). The number of viable cells was determined with WST-1 cell viability reagent (Roche Diagnostics, Mannheim, Germany) 24, 48 and 72 hours after transfection, as described previously [33] or the number of the cells was determined 72 hours after transfection by counting the cells in 96-well plate wells (n=8).

### Apoptosis assay

cSCC cells were transfected with control siRNA and AIM2 siRNAs (75nM). Apoptotic cells were detected 48 hours after transfection with In Situ Cell Death Detection Kit (Roche). Number of TUNEL positive cells was counted from three parallel image fields using 10× objective and compared to total cell number visualized by Hoechst 33342 (Invitrogen).

### Invasion assay

After transfection with AIM2 or control siRNA cSCC cell were incubated for 24 hours and seeded on ThinCert™ tissue culture inserts coated with collagen type I (Advanced Biomatrix, Fremont, CA) [29]. Nuclei were visualized by Hoechst 33342 (Invitrogen) after 48 hours and counted.

### Human cSCC xenografts

Human cSCC xenografts were established as previously [36]. Six-week-old severe combined immunodeficient (SCID) mice (CB17/Icr-*Prkdc^scid^*/IcrIcoCrl) (Charles River Laboratories) were randomly allocated into AIM2 (n=8) and control siRNA group (n=8). After 72 hours of transfection of highly tumorigenic cSCC cell line (UT-SCC7) with AIM2 or control siRNA, cSCC cells (5×10^6^) were injected subcutaneously into the back of mice. The size of tumors was measured twice a week and tumor volume was calculated with the formula V = (length × width^2^)/2 [38]. 21 days after the inoculation tumors were excised, embedded in paraffin and stained with hematoxylin and eosin (H&E). Proliferating cells were detected with anti-Ki-67 antibody (Dako, Glostrup, Denmark). Percentages of Ki-67-positive cells were determined by counting four distinct microscopic fields at 20× magnification (n=7-8). Effect of AIM2 knockdown on vascularization of the xenografts was assessed by IHC with anti-CD34 antibody (Santa Cruz Biotechnology, Santa Cruz, CA) [36]. Blood vessel formation was evaluated in each sample by counting the number of CD34-positive blood vessels in four randomly selected microscopic fields at 20× magnification.

### Statistical analysis

Mann-Whitney *U*-test and Student's *t*-test were used to analyze the significance of difference between groups. Fisher's exact test was used for analysis of IHC expression level between strong (+++) and moderate (++) expression as one group and weak (+) and absent (−) expression as the other group.

## SUPPLEMENTARY MATERIALS FIGURES AND TABLES



## Abbreviations

AIM2: absent in melanoma 2
AK: actinic keratosis
ASC: apoptosis-associated speck-like protein containing a caspase recruitment domain
cSCC: cutaneous squamous cell carcinoma
cSCCIS: cSCC in situ
EGF: epidermal growth factor
IFN: interferon
IFI16: interferon-inducible protein 16
IHC: immunohistochemistry
IL: Interleukin
KGF: keratinocyte growth factor
MMP: matrix metalloproteinase
NHEK: normal human epidermal keratinocyte
OTR: organ transplant recipient
qRT-PCR: quantitative real-time PCR
SCID: severe combined immunodeficient
TNF: tumor necrosis factor
TUNEL: Terminal deoxynucleotidyl transferase dUTP nick end labeling.
