# Supplementary material for: Tumor cell-specific AIM2 regulates growth and invasion of cutaneous squamous cell carcinoma
Source: Oncotarget. 2017 May 2;8(28):45825–36. doi: 10.18632/oncotarget.17573 (PMC5542230; doi:10.18632/oncotarget.17573)
Supplement: Supplementary file 1 [file oncotarget-08-45825-s001.pdf]

Tumor cell-specific AIM2 regulates growth and invasion of cutaneous squamous cell carcinoma

SUPPLEMENTARY MATERIALS

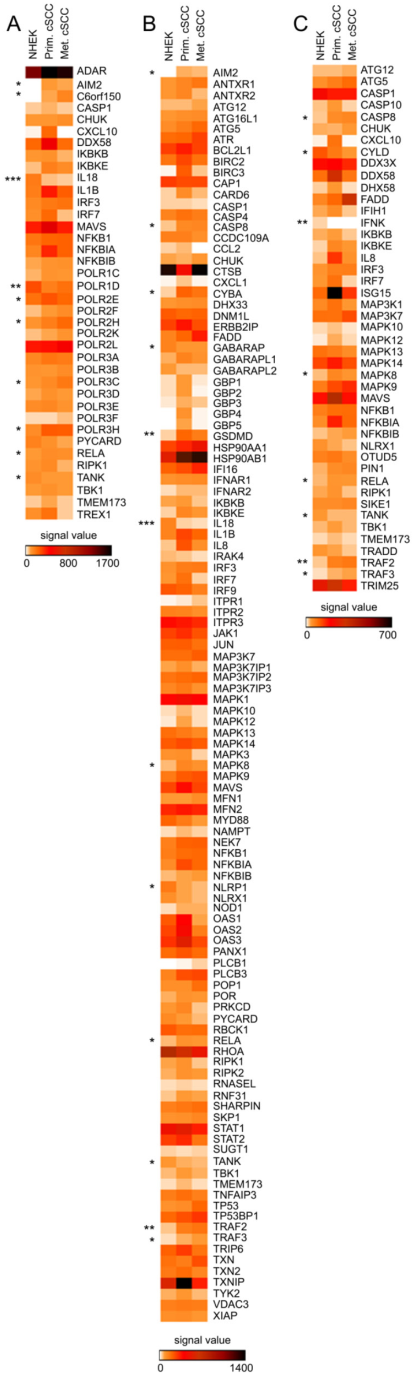

Supplementary Figure 1: The expression of inflammasome-related genes in NHEKs and cSCC cells. RNA-seq based analysis of the expression of genes involved in **(A)** cytosolic DNA-sensing, **(B)** NOD-like receptor signaling and **(C)** RIG-I-like receptor signaling KEGG pathways in primary (n=5) and metastatic (n=3) human cSCC cell lines and in NHEKs (n=5). *P<0.05, **P<0.01, ***P<0.001; Student's *t*-test.

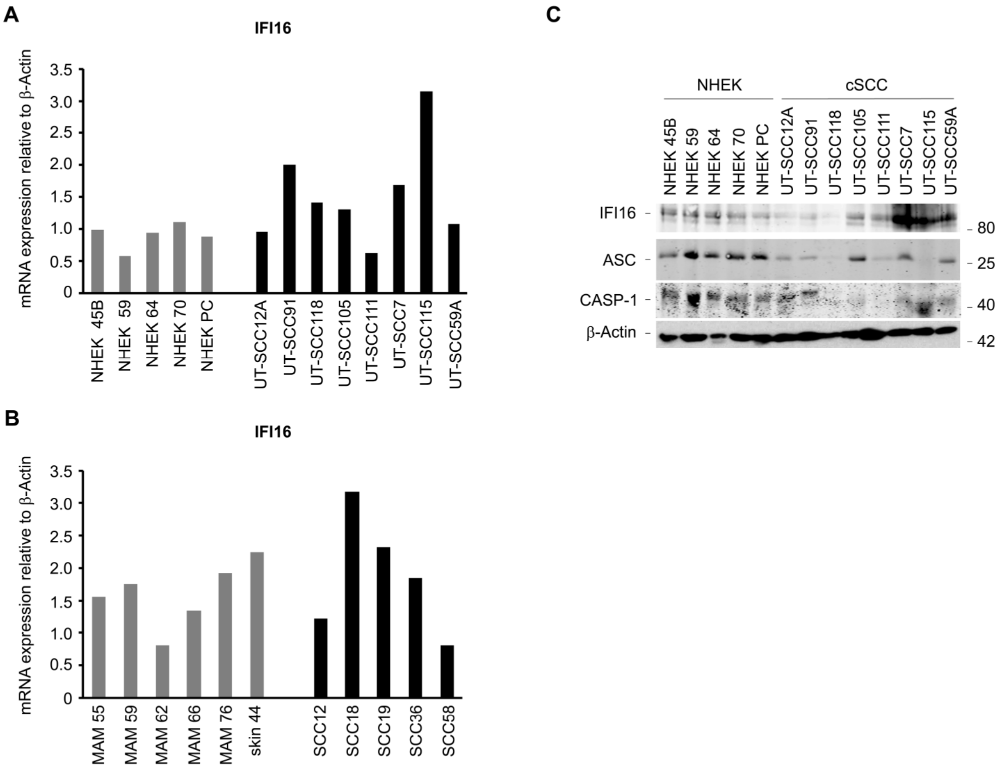

Supplementary Figure 2: Expression of IFI16, ASC and CASP1 in cSCC cells. (A) *IFI16* mRNA levels in cSCC cell lines (n=8) and NHEKs (n=5) were determined with qRT-PCR. **(B)** *IFI16* mRNA levels in cSCC tumors (n=6) and normal human skin (n=6) *in vivo* were analyzed by qRT-PCR. **(C)** IFI16, ASC and CASP1 protein levels in cell lysates of NHEKs and cSCC cells were determined by Western blotting with β-Actin as a loading control.

cSCC

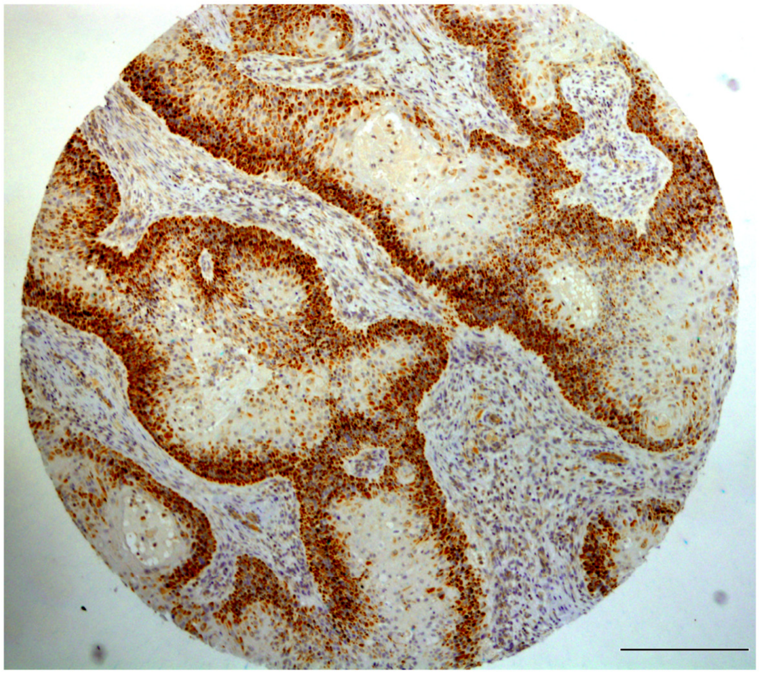

Supplementary Figure 3: Expression of AIM2 by tumor cells in cutaneous squamous cell carcinoma (cSCC). A TMA tissue section of cSCC was stained by IHC with anti-AIM2 antibody. AIM2 expression was detected in tumors cells at the invasive edges of the cSCC. Scale bar=500 µm.

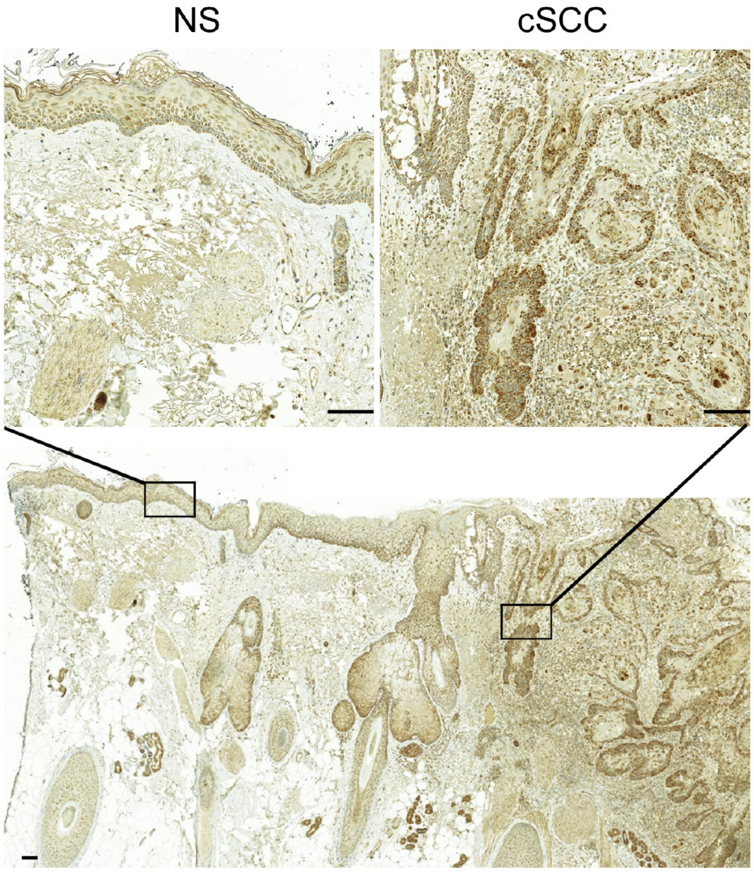

Supplementary Figure 4: Expression of AIM2 by tumor cells in cutaneous squamous cell carcinoma (cSCC). A whole tissue section of cSCC containing adjacent normal skin (NS) was stained by IHC with anti-AIM2 antibody. Specific expression of AIM2 was detected in tumor cells in cSCC. In the adjacent normal skin, expression of AIM2 was weak. Scale bar=100 µm.

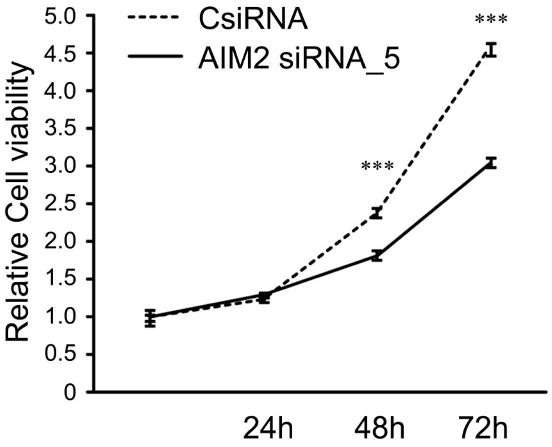

Supplementary Figure 5: Knockdown of AIM2 results in reduction in cSCC cell viability. The number of viable cSCC cells (UT-SCC7) was determined 24, 48, and 72 hours after transfection with AIM2 siRNA_5 and control siRNA (CsiRNA) (mean±SD, n=8). ****P*<0.001; Student's *t*-test.

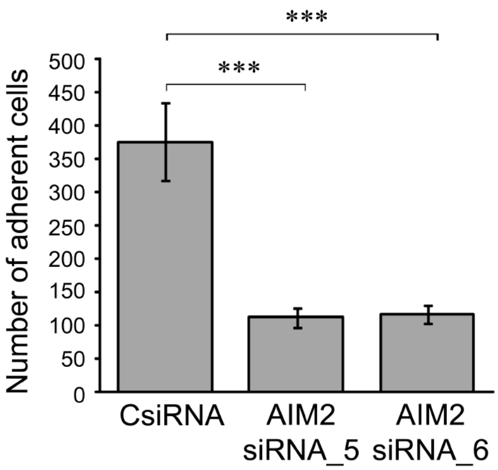

Supplementary Figure 6: Knockdown of AIM2 results in reduction in the number of cSCC cells. The number of cSCC cells (UT-SCC7) was counted 72 hours after transfection with AIM2 siRNA_5, AIM2 siRNA_6 and control siRNA (CsiRNA) (mean±SD, n=8). ***$P<0.001$; Student's *t*-test.

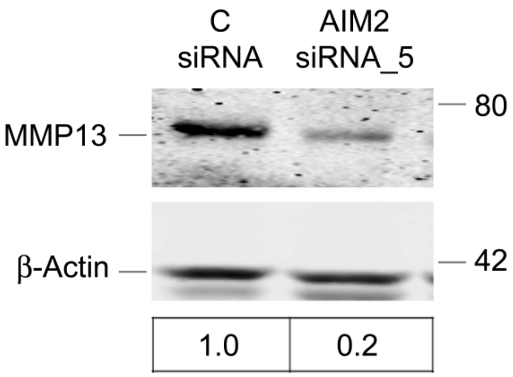

Supplementary Figure 7: Knockdown of AIM2 inhibits production of MMP13 in cSCC cells. Levels of MMP13 in conditioned media of cSCC cells were determined by Western blot analysis 72 hours after AIM2 knockdown with AIM2 siRNA_5. β-Actin in cell lysates was determined as a marker for equal loading. Levels of MMP13 were quantitated by densitometry and corrected for the level of β-actin. The values are shown relative to the level in culture transfected with control siRNA (C_siRNA).

Supplementary Table 1: Summary of networks associated with the differentially expressed molecules after AIM2 knockdown.

Top networks	Score[a]
Humoral Immune Response, Protein Synthesis, Cellular Growth and Proliferation	39
Embryonic Development, Tissue Morphology, Cell Cycle	34
Cellular Assembly and Organization, Connective Tissue Disorders, Developmental Disorder	32
Cell Death and Survival, Cellular Development, Organismal Development	32
Cancer, Endocrine System Disorders, Hematological Disease	30
Cardiovascular System Development and Function, Organismal Development, Tissue Morphology	26
Cellular Development, Cellular Growth and Proliferation, Embryonic Development	24
Cardiovascular System Development and Function, Organismal Development, Tissue Morphology	24
Cell Cycle, Cellular Assembly and Organization, DNA Replication, Recombination, and Repair	24
Nervous System Development and Function, Tissue Morphology, Cancer	23
Skeletal and Muscular System Development and Function, Cellular Assembly and Organization, Cellular Development	23
Cardiac Arrythmia, Cardiovascular Disease, Developmental Disorder	22
Post-Translational Modification, Cardiovascular Disease, Cell-To-Cell Signaling and Interaction	21
Embryonic Development, Immunological Disease, Inflammatory Disease	21
Cancer, Organismal Injury and Abnormalities, Respiratory Disease	21
Dermatological Diseases and Conditions, Developmental Disorder, Hereditary Disorder	19
Molecular Transport, Cellular Growth and Proliferation, Renal and Urological System Development and Function	19
Cellular Function and Maintenance, Small Molecule Biochemistry, Molecular Transport	19
Hereditary Disorder, Metabolic Disease, Developmental Disorder	19
Connective Tissue Disorders, Infectious Disease, Metabolic Disease	19
Nervous System Development and Function, Inflammatory Disease, Inflammatory Response	19
Cell Death and Survival, Cell Cycle, Cellular Movement	19
Cellular Compromise, Organismal Injury and Abnormalities, Skeletal and Muscular Disorders	18
Cell-To-Cell Signaling and Interaction, Cellular Movement, Hematological System Development and Function	17
Developmental Disorder, Hematological Disease, Hereditary Disorder	17

Cutaneous SCC cell lines (UT-SCC7, UT-SCC12A, and UT-SCC105) were transfected with AIM2 siRNA_6 or control siRNA (75 nM). 72 hours after transfection, whole transcriptome analysis was performed with RNA-seq. Summary of networks related to AIM2 knockdown identified by Ingenuity Pathway analysis (IPA) are shown ($P < 0.05$. FC log2 > 0.75)

[a]Derived from a *P* value indicating the likelihood that genes in a network are associated randomly
